# Supplementary material for: Deficits in Face Recognition and Consequent Quality-of-Life Factors in Individuals with Cerebral Visual Impairment
Source: Vision (Basel). 2023 Jan 25;7(1):9. doi: 10.3390/vision7010009 (PMC9944076; doi:10.3390/vision7010009)
Supplement: Supplementary file 1 [file vision-07-00009-s001.zip › vision-2135321-supplementary.pdf]

Supplemental Information

A. Selected Questions from the CVI Inventory

Face-related Questions

1. Do you (or does your child) find it difficult to recognize close relatives in real life?
2. Do you (or does your child) find it hard to recognize close relatives from photographs?
3. Do you (or does your child) mistakenly identify strangers as people known to them?
4. Do you (or does your child) have difficulty understanding the expression on your (or other people's) face?

Object-related Questions

5. Do you (or does your child) have difficulty naming common colors?
6. Do you (or does your child) have difficulty naming basic shapes, such as squares, triangles, and circles?
7. Do you (or does your child) find it hard to recognize well-known objects such as the family car, classroom door, etc.?
8. Do you (or does your child) find it hard to recognize an object if it is partially hidden or viewed from an unusual angle (such as a shoe under the bed with only the toe showing)?
9. Do you (or does your child) find it hard to recognize people, words, or objects if they are changed in any way, for example font of text, hairstyle?

The Five Questions

10. Do you (or does your child) find copying words or drawing time-consuming and difficult?
11. Do you (or does your child) have difficulty walking down stairs?
12. Do you (or does your child) have difficulty seeing things which are moving quickly, such as small animals?
13. Do you (or does your child) have difficulty seeing something which is pointed out in the distance?
14. Do you (or does your child) have difficulty locating an item of clothing in a pile of clothes?

B. Additional statistical analyses

1. <u>Unadjusted Repeated Measures</u>

Threshold

There was a significant effect of task ($F(1, 31) = 25.37$, $p < 0.0001$) and group ($F(1, 31) = 5.38$, p = 0.0.0271) on threshold. There was also a significant interaction between task and group ($F(1, 31) = 5.42$, $p = 0.0266$). Specifically, CVI was associated with a significant increase in blur threshold compared to controls for the faces task ($t(31) = 3.29$, $p = 0.0025$, adj. $p = 0.01$). There was no group difference in threshold for the glass pattern tasks ($t(31) = 0.01$, $p = 0.9949$, adj. $p = 1$).

Within the CVI group, there was a significant difference in threshold between tasks ($t(31) = 3.70$, p = 0.0.0008, adj. $p = 0.0032$), while there was an increased threshold for threshold within the control group for the faces task compared to the glass pattern task ($t(31) = 12.41$, $p < 0.0001$, adj. $p < 0.0001$).

Proportion Correct

There was an overall significant effect of task ($F(1, 31) = 24.21$, $p < 0.0001$) and group ($F(1, 31) = 5.67$, $p = 0.0007$) on proportion correct, with a significant interaction between task and group ($F(1, 31) = 6.33$, $p = 0.0173$). Specifically, CVI was associated with a significant reduction in the proportion correct compared to controls for the faces task ($t(31) = -3.30$, $p = 0.0024$, adj. $p = 0.0096$), but not for the glass pattern task ($t(31) = -0.86$, $p = 0.3946$, adj. $p = 1$).

Within both groups, participants successfully identified the target face more often than they did the glass pattern (CVI: $t(31) = 1.42$, $p = 0.1644$, adj. $p = 0.6576$; control: $t(31) = 6.94$, $p < 0.0001$, adj. $p < 0.0001$), although this did reach statistical significance for the CVI group.

Response time

There was an overall significant effect of task ($F(1, 31) = 64.42$, $p < 0.0001$) on response time, with an additional significant interaction between task and group ($F(1,31) = 15.87$, $p = 0.0004$). There was no overall significant effect of group on response time ($F(1, 31) = 0.08$, $p = 0.7778$). Specifically, CVI was associated with a trend of an increase in response time for the faces task compared to controls ($t(31) = 1.64$, $p = 0.1101$, adj. $p = 0.4404$). There was a significant group difference for the response time for the glass pattern task, but this did not survive correction for multiple corrections ($t(31) = -2.15$, $p = 0.0399$, adj. $p = 0.1596$).

Within both groups, participants took longer to respond in the face task compared to the glass pattern task (CVI: $t(31) = 8.89$, $p < 0.0001$, adj. $p < 0.0001$; control: $t(31) = 2.74$, $p = 0.0101$, adj. $p = 0.0404$).

2. Group Differences in SDQ and Dutton Outcomes without covarying for age (nonparametric)

There was a significant increase in peer problems ($S = 312.5$, $Z = 2.0892$, $p = 0.0367$, adj. $p = 0.1835$), emotional problems ($S = 320$, $Z = 2.2935$, $p = 0.0218$, adj. $p = 0.109$), and internalizing score ($S = 328$, $Z = 2.5633$, $p = 0.0104$, adj. $p = 0.052$) in CVI compared to controls, but these did not survive correction for multiple comparisons. No significant group differences were observed for the total difficulties ($S = 310.5$, $Z = 1.9580$, $p = 0.0502$) and externalizing score ($S = 269.5$, $Z = 0.5774$, $p = 0.5637$).

Individuals with CVI had challenges on a significantly greater number of questions from the CVI inventory related to face recognition ($S = 400$, $Z = 4.3228$, $p < 0.0001$), object perception and recognition ($S = 403.5$, $Z = 4.8101$, $p < 0.0001$), and the five questions ($S = 514.5$, $Z = 4.5719$, $p < 0.0001$).
